# Management of unspecified abdominal pain and its complications: A case reports

**DOI:** 10.1016/j.ijscr.2025.111494

**Published:** 2025-06-09

**Authors:** Nadya Rahmatika, Soetojo Wirjopranoto, Yufi Aulia Azmi, Bagus Wibowo Soetojo, Antonius Galih Pranesdha Putra, Kevin Muliawan Soetanto

**Affiliations:** a Faculty of Medicine, Wijaya Kusuma University, Surabaya, Indonesia; b Department of Urology, Faculty of Medicine Universitas Airlangga – Dr. Soetomo General Academic Hospital, Surabaya, Indonesia; c Department of Health Sciences, University of Groningen, University Medical Center Groningen, Groningen, the Netherlands; d Departement of Orthopaedic and Traumatology, Faculty of Medicine, Universitas Airlangga, Indonesia; e Department of Immunology, Faculty of Medicine Siriraj Hospital, Mahidol University, Bangkok, Thailand

**Keywords:** Bladder rupture, sepsis, Mortality, Case report, Infection

## Abstract

**Introduction and importance:**

Acute abdomen after section cesarean (SC) is a case that can occur, and the diagnosis of the cause is very challenging. This case report describes the immediate diagnostic and management of unspecified abdominal pain on Day 13th post-SC, accompanied by complications.

**Case presentation:**

A 30-year-old woman with a history of SC on the 13th day came to the Emergency Room (ER) with abdominal pain and decreased consciousness. Focused Assisted Sonography in Trauma (FAST) and Kidney Ureter Bladder (KUB) X-ray as a diagnostic tool was performed, followed by laparotomy as definitive management. We found a 7 cm bladder rupture in the bladder dome, 8 L of urine mixed with pus, and 300 cc of blood clots. We manually evacuated the urine and blood clots and refreshed the bladder tissue and cystostomy, and then the bladder was sutured. Double antibiotics were given for *Staphylococcus hemolyticus* infection.

**Clinical discussion:**

The diagnosis of abdominal pain was challenging. Late diagnosis can cause complications and mortality. FAST and KUB X-rays are useful tools in the care of bladder rupture after SC. Laparotomy is an option for treatment. In this case, the patient underwent laparotomy followed by cystostomy. The role of double antibiotics is necessary in cases of sepsis.

**Conclusion:**

Patients with unspecified abdominal pain after SC need immediate diagnosis. Diagnosis with FAST is sufficient to be done in unstable patient conditions. Immediate management with Laparotomy and Cystostomy combined with antibiotics can minimize mortality.

## Highlights

• Unspecified abdominal pain caused by bladder rupture can occur as a complications after section cesarean.
• Delay in recognizing and treating abdominal pain can lead to complications and mortality in patients.
• FAST, laparotomy exploration, and comprehensive post-operative care are the treatments.
• Cystostomy is used to reduce the workload of the bladder after bladder repair.

## 1 Introduction

Post-section cesarean (CS) discomfort, caused by surgical tissue injury, is a major source of patient dissatisfaction [1]. One of these problems can occur due to physiological or pathological causes. One of the problems that can arise is an acute abdomen after SC. Acute abdominal discomfort is a common presenting ailment in the Emergency Department (ED). Making an accurate diagnosis can be challenging. It becomes considerably more difficult and convoluted in the postpartum period, when other less prevalent but significant disorders must be evaluated [2]. Acute postoperative pain following SC is a significant issue for mothers who give birth after SC, necessitating the development of additional pain management techniques [3].

One of the differential diagnoses in acute abdomen after SC is the possibility of internal bleeding and bladder rupture. The anterior organ nearest to the uterus is the bladder. The most prevalent type of injuries sustained during obstetric or gynecological surgery are urological ones, with the bladder suffering the most damage. [4]. About 0.2 % of cases of original SC and 0.6 % of cases of recurrent CS involve bladder damage [5]. The bladder is the most often injured organ in obstetric/gynecologic surgeries such as cesarean section and hysterectomy, with an incidence of 13.8 occurrences per 1000 procedures [6]. Bladder injuries occur at different rates depending on the method and surgical approach. Bladder injuries have been found to have an incidence of 0.44 % with cesarean delivery [7].

Diagnosing bladder rupture might be difficult. It is an uncommon illness with a high morbidity and fatality rate. Ignoring a bladder rupture can lead to organ failure, sepsis progression, and even death [8]. Failure to diagnose bladder injuries can lead to consequences such as sepsis, ileus, and peritonitis [9]. Injuries to the bladder that could cause sepsis need to be treated surgically right away [10].

A computed tomography (CT) cystogram, retrograde cystography, or surgical exploration can all be used to detect bladder injuries following SC [11]. Retrograde cystography is a valuable diagnostic method for postoperative patients with suspected urologic damage. Abdominal CT with cystography has a strong diagnostic value in acute abdomen. Diagnostic laparotomy should always be considered if intraperitoneal bladder damage is suspected [12]. A FAST (focused assessment with sonography in trauma) can be used to detect the problem [13].

This case report describes immediate diagnostic and management of unspecified abdominal pain on Day 13th post-SC, accompanied by complications. It has been reported in line with the SCARE Guideline [14].

## 2 Case presentation

**Fig. 1:**
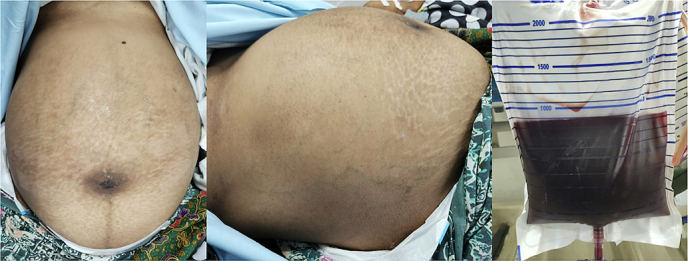
Clinical picture of the patient and gross hematuria.

**Fig. 2:**
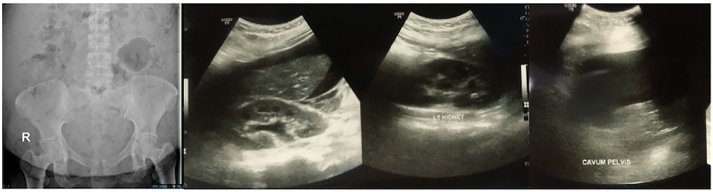
Radiology Examination, KUB X-ray, and FAST Results.

**Fig. 3:**
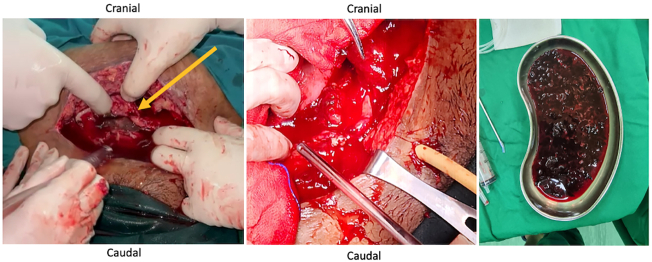
Durante the Operation, bladder repair and blood clot evacuation.

A woman, 30 years old, with a history of Section Cesarean (SC) on postoperative day 13th^,^ came to the emergency room (ER) of a tertiary referral hospital with chief complaints of abdominal pain and decreased consciousness. She was referred by the public health center from a small island. There was an enlarged abdomen since day 8th post-SC, followed by abdominal pain, shortness of breathing, gross hematuria, fever, loss of appetite since 2 days before hospital admission, vomiting (+), and nausea (+). The patient showed a decrease in consciousness since one day before hospital admission. This patient was referred to the hospital late due to bad weather conditions: heavy rain and strong winds on the islands, so that no ships could sail. Vital Signs showed Glasgow Coma Scale (GCS) 2 × 4, Blood Pressure (BP) 147/ 95 mmHg, heart rate 120 beats/ min, respiratory rate 26 breaths/ min, and temperature 38.5 °C. Physical Examination showed abdominal distention, bowel sound (+), and scar post-op (+). Urine Production: 1000 cc/ 24 h Hematuria (+) (Fig. 1). Laboratory Examination Results showed hemoglobin: 6.4 g/dL. Leucocyte: 35.89/ uL, albumin: 2.87, creatinine serum: 5.3 mg/ dL. Radiology Examination showed Kidney Ureter Bladder (KUB) X-ray: gas shadow (+), Focused Assisted Ultrasonography in Trauma (FAST): Bilateral mild hydronephrosis (+), balloon catheter (+) intra bladder, bladder not sufficiently filled with fluid (Fig. 2). We inserted a 20 fr with 3 way catheter to monitor the patient's total urine production. The patient underwent a blood transfusion before surgery and after surgery, 1 bag packed red cell (PRC)/ day. An empirical antibiotic, ampicillin 1 g/ 6 h intravenous (IV), and Metronidazole 500 mg/ 8 h intravenous (IV) were given to avoid the progression of sepsis. The patient underwent a laparotomy procedure. We re-opened the scar post-SC, layer by layer, and a bladder rupture was found to be 7 cm at the bladder dome. Also, 8 L of urine mixed with pus and a 300 cc blood clot intra-abdomen (Fig. 3). Those findings were evacuated. Then, there was a bladder evaluation; there was no other bladder rupture. We repaired the bladder, first refreshment the bladder tissue, and placed 20 fr catheters as a cystostomy. A cystotomy was performed because the bladder rupture >5 cm and irregular pattern. Then we sutured the bladder with 2 layers: the first layer with plain catgut 2.0 with continuous sutures, and then the second layer with Vicryl 2.0 with interrupted sutures. The intraperitoneal drain was inserted, followed by a 24 fr urethral catheter. Postoperative care was a blood transfusion of PRC 1 pack/ day until hemoglobin 10 g/dL (1 bag PRC/ day for 4 days). We changed the antibiotic when the blood and urine culture results showed *Staphylococcus hemolyticus*, sensitive to cefoperazone sulbactam 1 g/ 12 h IV combined with metronidazole 500 mg/8 h IV. The intraperitoneal drain was removed on the fifth postoperative day 5th before the patient was discharged from the hospital. The urethral catheter was removed on day 14th, and the cystostomy was removed on day 21st. Radiological follow-up was performed on the 30th day after cystostomy removal with cystography; no contrast leakage from the bladder, and there was no complaint about urine incontinence or other problems.

## 3 Discussion

Acute abdomen, in this case, is suspected to be caused by bladder rupture because there is a history of SC. Physical and supporting examinations are important in emergency conditions to establish a diagnosis. Predisposing variables for bladder injury during cesarean section include advanced labor, scarred uterus, intraabdominal adhesion, prolonged labor, and cesarean hysterectomy [15]. Bladder adhesions from prior abdominal surgeries are the primary risk factor for bladder damage during cesarean delivery [12]. Although abdominal or pelvic trauma is the most frequent cause of bladder rupture, it can also occur spontaneously or as a result of endoscopic or surgical treatments [16]. Cesarean sections are a major cause of iatrogenic bladder damage, and there is a link between the two procedures. Due to variables like adhesions and changed pelvic anatomy, cesarean sections—especially repeat surgeries and emergencies—increase the risk of bladder injury [7].

Supporting radiology, KUB X-ray, and FAST are sufficient in emergency conditions. Focused assessment with sonography for trauma (FAST) is a useful tool in the care of trauma patients, detecting free intra-abdominal and pericardial fluid [17]. The FAST assesses three possible areas within the peritoneal cavity for pathological fluid [18]. Focused Assessment with Sonography for Trauma (FAST) has emerged as a point-of-care ultrasound method [19]. Another method is KUB, a plain abdomen X-ray, often known as a KUB (kidneys, ureters, bladder), which can aid in the diagnosis of bladder injury. However, a CT scan may be more effective in identifying bladder injury [20].

Management in this case was carried out by laparotomy after the cause was confirmed (in this case, intraperitoneal bladder rupture, showed a free fluid from FAST) to prevent further complications. Patients in good condition without symptoms of sepsis or peritonitis may be candidates for conservative treatment with a urinary catheter for a month. [21]. A bladder rupture that is well-repaired has a very positive prognosis since the organ's blood supply is adequate to promote rapid and full healing [22]. In this surgery, the injured bladder wall is sutured. Urine leakage into the abdominal cavity during an intraperitoneal bladder rupture raises the risk of intraabdominal sepsis and necessitates prompt surgical repair [23]. In this case, the mixed urine with pus and blood clots was manually evacuated because they were the source of infection and caused sepsis to decrease consciousness.

The patient, in this instance, underwent an open cystotomy. During a cystostomy surgical operation, a bladder hole is made so urine can pass via a catheter inserted into the abdominal wall. It can help individuals who cannot urinate regularly, temporarily, or permanently. A cystostomy may be necessary for several causes, such as damage or injury to the urethra, obstruction of the bladder, and other illnesses that affect the kidneys or urinary tract. During an open cystotomy, the pubic symphysis is covered by a small incision [16].

In this case, it is important to do bladder tissue refreshment, cystostomy, intraperitoneal drain, and a large caliber 24Fr urinary catheter. Refreshment bladder tissue is done so that bad tissue is removed, which will accelerate bladder healing. An intraperitoneal drain is used to remove residual fluid from the intraperitoneal space, a large urethral catheter is used, and a cystostomy is performed to rest the bladder. Proper wound healing happens in three stages, allowing the injury to heal and new tissue to replace it. The three steps are inflammation, proliferation, and maturation. [24].

The role of double antibiotics in this case is because it is accompanied by sepsis. The patient has switched to antibiotics. The patient initially received metronidazole and ampicillin. After a culture revealed Staphylococcus hemolytic, the antibiotic was switched to Cefoperazone sulbactam with Metronidazole. Patients with sepsis necessitate sophisticated bedside decision-making regarding when medicines are recommended rapidly and which regimens are appropriate [25]. The majority of *Staphylococcus haemolyticus* makes up the human skin microbiome. It is pervasive among medical personnel and in hospitals, leading to the emergence of a bacterium that causes nosocomial infections [26].

In this case, preventive measures are necessary. Preventing bladder rupture after an SC involves meticulous surgical methods, prior planning, and rapid diagnosis and repair of damage [4]. Bladder injury during cesarean section is not uncommon today (during primary cesarean section, 0.2 %, and repeat cesarean section, 0.6 %). Recognition of the injury during surgery and prompt repair result in the best outcomes. Appropriate preoperative counseling and good operative records can help avoid litigation [15]. The patient requires follow-up because it is included in a complex bladder rupture. Cystography is performed to assess bladder wall healing after complex injury repair or if there are risk factors for poor wound healing [27]. This case report describes immediate diagnostic and management of unspecified abdominal pain after SC, accompanied by complications that can impact future health services. Further studies are expected to increase the sample size and conduct more in-depth clinical studies.

## 4 Conclusion

Patients with unspecified abdominal pain after cesarean section need immediate diagnosis. Diagnosis with FAST is sufficient to be done in unstable patient conditions. Immediate management with Laparotomy followed by cystostomy, combined with antibiotics can minimize mortality.

## Ethical approval

Ethical approval for this study was provided by Health Research Ethics Committee of the hospital.

## Author contributions

Nadya Rahmatika: Conceptualization, Data Curation, Writing-Original draft preparation

Bagus Wibowo Soetojo: Conceptualization, Data Curation, Writing-Original draft preparation

Soetojo Wirjopranoto: Conceptualization, Methodology, Data Curation, Investigation, Writing-Original draft preparation, Supervision, Validation

Yufi Aulia Azmi: Conceptualization, Methodology, Data Curation, Investigation, Writing-Original draft preparation, Supervision, Validation

Antonius Galih Pranesdha Putra: Writing-Original draft preparation, Writing-Reviewing, and Editing

Kevin Muliawan Soetanto: Writing-Original draft preparation, Writing-Reviewing, and Editing

## Funding statement

The author(s) received no external financial support.

## Declaration of competing interest

The authors declare no conflict of interest.
